# Meeting abstracts from the Annual Conference “Clinical Genetics of Cancer 2023”

**DOI:** 10.1186/s13053-024-00294-4

**Published:** 2024-11-12

Edited by Jan Lubiński

The Supplement Editor declares no competing interests

## A1 Blood copper and arsenic levels and the occurrence of colorectal cancer

### Piotr Baszuk^1,2,^, Paulina Stadnik^1^, Wojciech Marciniak^1,2^, Róża Derkacz^1,2^, Anna Jakubowska^1^, Cezary Cybulski^1,2^, Tomasz Huzarski^1,2,3^, Jacek Gronwald^1,2^, Tadeusz Dębniak^1^, Katarzyna Białkowska^1^, Magdalena Muszyńska^1,2,3^, Sandra Pietrzak^1^, Józef Kładny ^4^, Rodney J. Scott ^5,6,7^, Steven A. Narod^8,9^, Jan Lubiński^1,2^, Marcin R. Lener^1^

#### ^1^International Hereditary Cancer Center, Department of Genetics and Pathology, Pomeranian Medical University in Szczecin, Szczecin, Poland; ^2^ Read-Gene, Grzepnica, Poland; ^3^Department of Clinical Genetics and Pathology, University of Zielona Góra, Zielona Góra, Poland; ^4^Department of General Surgery and Surgical Oncology, First Clinical Hospital of Pomeranian Medical University in Szczecin, Szczecin, Poland; ^5^Priority Research Centre for Cancer Research, Innovation and Translation, Hunter Medical Research Institute, New Lambton Heights, Australia; ^6^School of Biomedical Sciences and Pharmacy, Faculty of Health and Medicine, University of Newcastle, Australia; ^7^Division of Molecular Medicine, Pathology North, John Hunter Hospital, New Lambton, Australia; ^8^Women's College Research Institute, Toronto, Canada; ^9^Dalla Lana School of Public Health, University of Toronto, Toronto, Canada

*Hereditary Cancer in Clinical Practice* 2024, **22(1):**A1

Colorectal cancer is one of the most common types of cancer in both men and women. There are several approaches to detect early colorectal cancer including testing relevant markers in stool or blood (proteins; DNA; mRNA and long non-coding RNA; microRNA; metabolites, gut microbiome and platelets) as well as algorithms using machine learning techniques. Invasive methods of early detection of colorectal cancer include: colonoscopy, sigmoidoscopy and colonography based on computed tomography. There is a need to identify new markers for early detection of colorectal cancer. The influence of elements and inherited changes in genes on cancer risk and incidence has been one of the issues studied in the last decades.

In this study we examined whether blood arsenic and/or copper levels combined with specific polymorphisms can be used as a marker for colorectal cancer detection.

A retrospective case-control studies were performed among 187 colorectal cancer patients and 187 matched controls. All participants provided written informed consent to be enrolled into the following study. Information about: age, sex, smoking status and familial aggregation of cancer was obtained from all participants. All diagnosed participants were asked about additional clinical data related with colorectal cancer. From each study participant pre-treatment peripheral blood was collected for arsenic and copper level measurements using inductively coupled-plasma mass spectrometry (ICP-MS) and for genotyping. Selected variants in ten genes were genotyped: rs13181 in *ERCC2*, rs1799782 in *XRCC1*, rs7191779 in *MT1B*, rs1695 in *GSTP1*, rs2032582 in *ABCB1*, rs1800566 in *NQO1*, rs12915189 in *CRTC3*, rs1050450 in *GPX1*, rs4880 in *SOD2*, and rs1001179 in *CAT*.

According to the study results – a low blood arsenic level (0.27–0.67 µg/L) was associated with an increased frequency of colorectal cancer among women (OR: 3.69; *p* = 0.005). This correlation was significantly greater among women carrying functional polymorphisms: *CAT* rs1001179 – nonCC (OR:19.4; *p* = 0.001); *ABCB1* rs2032582 – CC (OR:14.8; *p* = 0.024); *GPX1* rs1050450 – CC (OR: 11.6; *p* = 0.002) and *CRTC3* rs12915189 – nonGG (OR: 10.3; *p* = 0.003). As a result of the research performed, it was also observed that high blood copper level (931–2 043 µg/L) is associated with an increased incidence of colorectal cancer (OR: 12.7; 95% CI: 4.98–32.3; *p* < 0.001). This correlation was significantly greater among study participants carrying particular gene variants: *ABCB1* rs2032582–nonCC (OR: 33.7; 95% CI: 4.04–281; *p* = 0.001); *MT1B* rs7191779–nonGG (OR: 16.6; 95% CI: 3.32–83.4; *p* < 0.001); *CAT* rs1001179–CC (OR: 16.1; 95% CI: 3.68–70.7; *p* < 0.001); *SOD2* rs4880–nonGG (OR: 15.9; 95% CI: 3.27–77.0; *p* < 0.001); *GSTP1* rs1695–nonAA (OR: 15.9; 95% CI: 1.54–164; *p* = 0.02) and *XRCC1* rs1799782–CC (OR: 15.6; 95% CI: 5.00–48.5; *p* < 0.001).

Based on above studies it can be stated that blood arsenic and copper level measurements can be valuable marker in order to select patients for further colorectal cancer diagnostics. Selection effect for colorectal cancer diagnosis can be stronger among individuals with particular gene polymorphisms.

## A2 Germline mutations associated with acute myeloid leukemia

### Aneta Bąk^1^, Katarzyna Skonieczka^1^, Anna Jaśkowiec^2^, Anna Junkiert-Czarnecka^1^, Marta Heise^1^, Maria Pilarska-Deltow^1^, Stanisław Potoczek^2^, Maria Czyżewska^3^, Olga Haus^1^

#### ^1^Department of Clinical Genetics, Collegium Medicum in Bydgoszcz,Nicolaus Copernicus University in Toruń, Toruń, Poland; ^2^Department of Hematology, Blood Neoplasms and Bone Marrow Transplantation, Medical University in Wrocław, Wrocław, Poland; ^3^Department of Hematology, Municipal Hospital in Toruń, Toruń, Poland

*Hereditary Cancer in Clinical Practice* 2024, **22(1):**A2

https://hccpjournal.biomedcentral.com/articles/10.1186/s13053-021-00200-2

## A3 Different histological types of breast cancer share common epigenetic and transcriptomic signature that predicts clinical outcome

### Jan Bińkowski, Tomasz Kazimierz Wojdacz

#### Independent Clinical Epigenetics Laboratory, Pomeranian Medical University in Szczecin, Szczecin, Poland

##### **Correspondence:** Tomasz K. Wojdacz, (tomasz.wojdacz@pum.edu.pl)

*Hereditary Cancer in Clinical Practice* 2024, **22(1):**A3

**Background**

Breast cancer is the leading cancer diagnosis in women accounting for over 10% of new cancer cases annually. It is also the most frequently diagnosed malignant tumor and primary cause of cancer deaths in women globally. Despite substantial improvement in the understanding of breast cancer biology, we are still deciphering the contribution of methylome changes to breast cancer pathology and association of those changes with expression of genes involved in cancer development. In our study we analyzed methylomics and transcriptomics profiles of three different breast cancer types: infiltrating duct carcinoma (*n* = 50), breast lobular carcinoma (*n* = 50) and mucinous adenocarcinoma (*n* = 14)) as well as healthy breast tissue (*n* = 46) to assess whether there is methylation signature common for those different cancer types associated with the gene expression changes observed during breast cancer pathology.

**Methods**

Statistical analyses were conducted using eDAVE platform that we recently developed [1] and Kaplan-Meier Plotter [2] tool. Gene set enrichment analyses as well as protein-protein interactions networks were performed using FUMA [3] and STRING [4] tools respectively.

**Results**

We found a subset of 615 genes and 13714 CpG sites with statistically significant aberrant expression (FDR ≤ 0.05, log_2_(FC) > 2) and methylation levels (FDR ≤ 0.05, |delta|> 0.1) respectively, between cancer and healthy breast tissue, that were common for all analyzed cancer types. The subset of 171 of the identified genes harbored aberrantly methylated CpG sites. The Gene Set Enrichment Analyses (GSEA) based on this subset of genes confirmed that expression of those genes is specific for adipose and breast mammary tissues (FDR ≤ 0.05) and those genes are significantly enriched in molecular pathways involved in: epithelial-mesenchymal transition, estrogen response as well as Wnt signaling. Survival analysis based on over 2000 breast cancer samples deposited in the TCGA, GEO and EGA databases showed that expression of 10 of the identified genes is significantly (*p*-value <  = 0.05) associated with patients relapse free survival as well as overall survival.

**Conclusion**

Our study showed that there is common methylation signature associated with transcriptome of breast cancer and uniformly shared between histologically different types of this cancer. The identified methylation signature is furthermore strongly associated with the expression changes of genes involved in breast cancer pathology and strongly predicts clinical outcomes of patients such as overall survival and relapse free survival.

**Funding**

This study was funded by Polish Returns grant program from Polish National Agency for Academic Exchange, grant ID: PPN/PPO/2018/1/00088/U and OPUS22 grant from National Science Centre, grant ID: 2021/43/B/NZ2/02979.

**References**

1. Bińkowski J, Taryma-Leśniak O, Sokolowska KE, Przybylowicz PK, Staszewski M, Wojdacz TK: eDAVE – Extension of GDC data analysis, visualization, and exploration tools. Computational and Structural Biotechnology Journal 2023, 21:5446-5450.
2. Győrffy B: Survival analysis across the entire transcriptome identifies biomarkers with the highest prognostic power in breast cancer. Comput Struct Biotechnol J 2021, 19:4101-4109.
3. Watanabe K, Taskesen E, van Bochoven A, Posthuma D: Functional mapping and annotation of genetic associations with FUMA. Nature Communications 2017, 8(1):1826.
4. Szklarczyk D, Gable AL, Lyon D, Junge A, Wyder S, Huerta-Cepas J, Simonovic M, Doncheva NT, Morris JH, Bork P et al: STRING v11: protein-protein association networks with increased coverage, supporting functional discovery in genome-wide experimental datasets. Nucleic Acids Res 2019, 47(D1):D607-d613.

## A4 Hereditary diffuse type gastric cancer: CDH1—An update and a glimpse of preliminary science

### Tanya Bisseling

#### Expert Center for Hereditary Cancer, Radboudumc Nijmegen, Netherlands

*Hereditary Cancer in Clinical Practice* 2024, **22(1):**A4

Hereditary Diffuse type Gastric Cancer (HDGC) is a very rare condition that affects about 1:10,000-1:20,000 live births. In fact HDGC is a syndrome and it is associated with an increased lifetime risk for diffuse gastric cancer and lobular breast cancer. Additionally a cleft lip is common. Due to its rarity HDGC is underrecognized and development of translational and clinical insight proceed slowly. In this update you will learn the current knowledge on HDGC. Additional insight in latest scientific developments.

## A5 Mirror results in population screening of breast and colorectal cancer in Lithuania

### Pavel Elsakov

#### State Research Institute, Innovative Medicine Center, Vilnius, Lithuania

*Hereditary Cancer in Clinical Practice* 2024, **22(1):**A5

The mortality rates are high in Lithuania with relatively low incidence such as of Colorectal and Breast cancers. For intense in Lithuania the CRC age-standardized incidence rate is 23.4 and mortality rates 13.7 while in the Netherland comparative rates are 40.2 and 13.4 respectively. Similar the Breast cancer incidence rate in Lithuania is 65.2 and mortality rates 23.4 in the Netherland comparative rates are 131.3 and 26 respectively. The patient’s survival in case of early detection of the cancer by FOBT screening and colonoscopy for colorectal cancer and mammography screening for breast cancer is an indicator of mortality from these cancers. In Lithuania 2009 started a screening program using the FIT (OC-Sensor, Japan) with automated reading techniques. The target population of study according to criteria in age 50-74 years for potential screening was 45 330 subjects: female 27 909(61.6%) and 17 421(38.4%) male. Patients whose samples revealed an FIT value Hb > 100 –ng/ml of buffer underwent colonoscopy. The participation rate in screening calculated every 2 years 1-4 round was 33.6%, 35.1%, 40.2%, 23.7%. From 35 689 participated in screening patients in 176(0.49%) was diagnosed CRC cases and from 9 641 non-participated patients was diagnosed 94(0.98%) CRC cases. Distribution of CRC by stage I-IV and ten years survival of screened and non-screened patients were not significantly different *P* = 0.13 and *P* = 0.4 respectively. Results of these study we compare with mammography screening program results for Breast cancer which started in Lithuania 2005 [Steponaviciene et al., 2019]. According to program women 50-69 years old was tested by mammogram every 2 years. The target population for screening 2016 January was 435.000. Only about half of the women who are recommended to participate in screening in mammography have participated. Screen-detected tumors represent only a small proportion (up to 28%) of tumors diagnosed in women aged 50-69 years. The distribution of diagnosed colorectal and breast cancer in screened patients by (TNM) stage was similar: Colorectal cancer Stage I-31.3%, II-30.7%, III-27.8%, IV-10.2%; Breast cancer – 46.3%, 34.2%, 14.1%, 3% respectively. Screening of average risk population is limited because the criteria of age are 50-74 for CRC and 50-69 years for breast cancer. A potential young below 50 years subjects with high risk to hereditary cancer including Lynch Syndrome and BRCA1/2 predisposition cancer are not accepted in screening and don’t impact on possible changes in improving of early diagnosis theses cancers. However in study 1993-97 of CRC patients under 55 years was suggested that a positive family history was not a clear indicator of early stage diagnosis. The Incidence Ratio of stage I-II for individual with positive family history for CRC was 0.95, whereas for individual for CRC with non – CRC family history it was 1.05. In 2009-2019 was introduced in medical practice a screen first colon cancer tumour by MSI and IHC staining according to histological criteria and young age below 50 years were diagnosed 54 suspected for Lynch syndrome patients (32 man and 22 women). Any one a CRC did not diagnosed in stage-I (TNM). The distribution of diagnosed CRC stage in each stage were: Stage II- 19(35.2%), III- 21(38.8%), and IV-10 (18.5%). In study of the familial and non-familial 263 breast cancer patients (mean age 30-86) with the founder BRCA1 mutation carrier status identified between 1996-2009 distribution diagnosed cancer cases by (TNM) stage were: Familial cancer stage I -27.7%, II-53.2%, III-19.1%; Non familial cancer I-35.6%, II-45.4%, III-19.0% respectively. Distribution of cancer patients with mutation was: Stage I -26.1%, II-65.2%, III-8.7%.

Conclusions: The participation rate for CRC and Breast cancer in screening was less than EU guideline set minimum 45% and 70% respectively. It did not improve a detection of CRC by stage *P* = 0.13 and ten years survival of screened and non-screened of CRC patients is not significantly different *P* = 0.39. Mirror results show and breast screen-detected tumors represent only a small proportion (up to 28%) including tumor in Stage I -46.3%.

## A6 Germline variants in cancer patients with personal and family history of colorectal cancer

### Asta Försti^1,2^

#### ^1^Hopp Children's Cancer Center (KiTZ), Heidelberg, Germany; ^2^Division of Pediatric Neurooncology, German Cancer Research Center (DKFZ), German Cancer Consortium (DKTK), Heidelberg, Germany

*Hereditary Cancer in Clinical Practice* 2024, **22(1):**A6

Multiple primary cancers in a single individual, as well as family history of the same cancer, are features of hereditary cancer. About 15% of colorectal cancer (CRC) patients have first-degree relatives affected by the same malignancy. However, for most families the cause of familial aggregation of CRC is unknown. To identify novel high-to-moderate-penetrance germline variants underlying CRC susceptibility, we performed whole genome sequencing (WGS) in germline DNA of Polish CRC patients with personal and family history of colorectal cancer. After WGS, we used in silico tools to evaluate the effect of the identified nonsynonymous and loss-of-function (STOPgain, frameshift and splice site) variants on the function of the corresponding proteins. We identified variants in genes known to be involved in CRC biology, chromatin and transcription regulation, general cellular processes and immune response. Our findings contribute to the identification of unrecognized genetic causes of familial CRC.

## A7 Genetic polymorphisms, response to treatment and adverse effects in breast cancer patients treated with FAC chemotherapy

### Ewa Grzybowska

#### Maria Skłodowska-Curie National research Institute of Oncology, Gliwice, Poland

*Hereditary Cancer in Clinical Practice* 2024, **22(1):**A7

https://www.ncbi.nlm.nih.gov/pmc/articles/PMC5823653/

## A8 Clinical significance of germline variants in *ASXL1*,* CHD1*,* IDH1*,* SETD2* and *TET2* epigenetic genes and their association with prostate cancer risk in Polish men – preliminary results

### Marta Heise^1^, Piotr Jarzemski^2^, Aneta Bąk^1^, Anna Junkiert-Czarnecka^1^, Maria Pilarska-Deltow^1^, Olga Haus^1^

#### ^1^Department of Clinical Genetics, Faculty of Medicine, Collegium Medicum in Bydgoszcz, Nicolaus Copernicus University in Toruń, Toruń, Poland; ^2^Department of Urology, Jan Biziel University Hospital in Bydgoszcz, Bydgoszcz, Poland

*Hereditary Cancer in Clinical Practice* 2024, **22(1):**A8

**Introduction**

The epigenetic variants are present in all human cancers and associated with genetic alterations to drive a cancer phenotype. Thus, we searched for germinal variants in *ASXL1*, *CHD1*, *IDH1*, *SETD2* and *TET2* epigenetic genes in Polish prostate cancer patients and controls and analyzed the impact of them on disease clinical course, including overall survival time.

**Material**

The material of investigation was DNA from 97 men with prostate cancer (PC) from all over Poland and DNA from 100 men—volunteers, healthy at the time of the study. The median age of patients at PC diagnosis was 60,4 ± 6,3 years (45–76). The mean age of controls was 59.9 ± 6.6 and matched the PC group (46 to 74).

**Methods**

NGS and Sanger sequencing.

**Results**

16 variants of *ASXL1*, *CHD1*, *IDH1*, *SETD2* and *TET2* epigenetic genes were detected in 14 PC patients. There were 9 missense variants (1 in *ASXL1*, 2 in*CHD1*, 1 in*IDH1*, 3 in*SETD2* and 2 in*TET2*), 1 duplication of *ASXL1* and 6 silent variants (2 in *CHD1* and 4 in*TET2*). All detected variants are localized in coding sequences of genes. Bioinformatic analysis of all variants was performed using Franklin or VarSome databases. Among detected variants, there were: 1 pathogenic, 1 likely pathogenic, 9 variants of uncertain significance (VUS), 1 likely benign and 4 benign. Two PC patients were carriers of two variants in different genes. The first of them was a carrier of *ASXL1* c.1934dupG pathogenic variant and *TET2* c.2370G > A variant of uncertain significance. The second was a carrier of *CHD1* c.2321A > T and *TET2* c.972A > G benign variants. 10 prostate cancer patients were carriers of pathogenic, likely pathogenic or VUS (P, LP or VUS) variants. 8 of 10 (80%) P, LP or VUS carriers and 54 of 87 (62,1%) non-carriers of investigated genes had at least one relative with cancer, including breast, uterus, stomach, colon, ovary, lung, larynx, kidney, liver, bladder, pancreas, lip, nose, blood, bone, duodenal cancers, glioblastoma and chronic lymphocytic leukemia (OR = 2.4, *p* = 0.3). 4 out of 10 P, LP or VUS carriers originated from families fulfilling HPC criteria and 6 out of 10 from families without HPC (PC frequency: 19,1% vs 7.9%, OR = 2.75, *p* = 0.14, trend). For the survival analysis, the patients were followed from the date of biopsy (confirmation of prostate cancer) until death, or in living patients, five-year survival was analyzed. 9 P, LP or VUS carriers diagnosed with PC between 2005 and 2007 survived five years from the date of biopsy and 1 carrier with PC diagnosed in 2019 is still living.

**Comment**

We found germline variants in each of the tested genes, but only 1 of them was pathogenic (*ASXL1* c.1934dupG) and 1 was likely pathogenic (*TET2* c.2218C > T). The frequency of both was 2,1% in the tested group and they were not detected in healthy men (*p* = 0.3). Noteworthy is a high OR (5.26) of disease occurrence in these patients. Additionally, the 9 variants of uncertain significance in *ASXL1* (c.3623C > T), *CHD1* (c.4949C > T, c.3723A > G, c.1434C > T), *IDH1* (c.565A > G), *SETD2* (c.3383C > G) and *TET2* (c.3930G > A, c.2370G > A, c.4161C > T) genes were detected. The presence of P, LP or VUS may be associated with hereditary prostate cancer, but this observation should be confirmed on larger PC groups. Due to the detection in Polish prostate cancer patients of a large number of epigenetic genes germline variants, including pathogenic, likely pathogenic and variants of uncertain clinical significance, there is a need to perform next investigations in this area and focus mainly on germline variants of genes involved in epigenetic processes.

## A9 Germline genetics of familial cancer

### Kari Hemminki^1,2^

#### ^1^European Research Area Chair of Translational Oncology, Charles University Medical School, Pilsen, Czech Republic; ^2^German Cancer Research Center, Heidelberg, Germany

*Hereditary Cancer in Clinical Practice* 2024, **22(1):**A9

The presentation will start with the common definitions of population genetic parameters with examples on how familial relative risks (FRRs) of cancer can be modeled in terms of genotype relative risk, population attributable fraction (PAF) and allele frequency. The cumulated results show that the common association studies on genes with minor allele frequency > 10% have power to detect disease-causing variants conferring PAFs > 10%, which can be compared to known genes, such as BRCA1 with an allele frequency of 0.1% and PAF of 1.8%. Yet, common low-risk variants confer low FRRs, typically of < 1.5. The models show that candidate gene studies may be able to identify genes conferring close to 100% of the PAF, but they may not explain the empirical FRRs. In order to explain FRRs, rare, high-penetrant genes or interacting combinations of common variants need to be uncovered. However, the candidate gene studies for common alleles do not target this class of genes. Next we review the estimated genetic basis of familial cancer as obtained from The Swedish Family-Cancer Database. The highest proportions of familial cancer were found for prostate (26.4%), breast (17.5%) and colorectal (15.7%) cancers. We discuss in more detail the germline genes found in these cancers and in further urological cancers. We conclude that the ever more detailed germline landscape of common cancers can be reasonably accommodated by the empirical family data on these cancers.

## A10 Early Insights from Hereditary Breast and Ovarian Cancer Risk Genetic Screening Pilot in Latvia

### Arvids Irmejs^1^, Zanda Daneberga^1^, Hildegunn Hoberg-Vetti^2^, Catherin Cathrine Bjorvatn^2^, Ilva Kononova^3^, Emil Syundyukov^3^, Ieva Danovska^3^, Agate Kalcenaua^3^, Martins Mednis^3^, Eva Pildegovica^3^, Edvins Miklasevics^1^, Janis Gardovskis^1^

#### ^1^Institute of Oncology, Riga Stradins University, Riga, Latvia; ^2^Haukeland University Hospital, Bergen, Norway; ^3^Longenesis Ltd, Riga, Latvia

*Hereditary Cancer in Clinical Practice *2024, **22(1):**A10

**Introduction**

Approximately 80% of *BRCA1/2* positive breast and ovarian cancers are diagnosed at stages II-IV. Despite the availability of modern treatments, the associated morbidity, mortality and costs remain significant. Therefore, the identification of *BRCA1/2* carriers in a presymptomatic stage is the ultimate goal to improve the prognosis for individuals at risk of hereditary breast and ovarian cancer. In many countries family cascade testing is of limited effectivity due to different reasons. The aim of this study is to evaluate the feasibility of implementing a hereditary breast and ovarian cancer risk genetic population screening program in Latvia.

**Material and methods**

The Institute of Oncology Riga Stradins University (RSUIO), in collaboration with private companies, conducted a pilot study utilizing the digital engagement tool, Longenesis Engage. Eligible women who had previously consented to participate in breast cancer risk assessment projects on skrinings.lv were invited via email, as well as through an email campaign initiated by Lindex. They accessed detailed information on a digital platform and provided consent digitally. Participants, females aged 25–59 without a personal cancer history, completed family cancer history assessments and psychological questionnaires (PHQ9 and GAD-7). Participants then downloaded a laboratory referral form to collect a saliva sample at the nearest private laboratory. Samples were sent to RSUIO for comprehensive BRCA1/2 testing. Negative results will be communicated digitally after completing follow-up questionnaires. In case of positive result, person will be contacted by phone and invited to visit clinical geneticist with following enrollment in the surveillance programme.

**Results**

Between September 4th and November 7th, 2023, a total of 3 438 invitation emails were sent as part of the research initiative. Of the recipients, 49% (1 686 out of 3 438) opened the email, and within this group, 54% (925 out of 1 686) visited the project's digital platform to access detailed project information. Of those who visited the platform, 64% (598 out of 925) provided informed consent, with the majority (86%) opting for a digital signature. By the end of the reporting period, 130 saliva samples had been received at the laboratory. This yields an overall response rate of 17% (598 out of 3 438) for the initial two months of the project.

**Conclusions**

The findings from this research initiative suggest that hereditary breast and ovarian cancer risk genetic population screening through digital communication channels is a viable approach. It is important to note that direct comparison with existing screening program percentages may not be straightforward due to the unique nature of this endeavour. While the results are promising, it is imperative to gather feedback and further refine the methodology.

Enhancements in email title and content, improved user-friendliness of the digital platform, targeted public relations efforts, and active involvement of family physicians and gynaecologists are potential strategies to augment the participation rate in future iterations of this screening program.

The study is supported by internal grants from Riga Stradins University, with additional funding provided by the private company Lindex. In-kind contributions have also been generously provided by the digital platform provider, Longenesis, and E. Gulbja laboratory.

## A11 Correlation between Se, Zn levels and survival among prostate cancer patients

### Sandra Pietrzak^1^, Wojciech Marciniak^2^, Róża Derkacz^2^, Milena Matuszczak^1^, Adam Kiljańczyk^1^, Piotr Baszuk^1^, Marta Bryśkiewicz^1^, Andrzej Sikorski^3^, Jacek Gronwald^1^,Marcin Słojewski^3^, Cezary Cybulski^1^, Adam Gołąb^3^, Tomasz Huzarski^1^, Tadeusz Dębniak^1^, Marcin Lener^1^, Anna Jakubowska^1^, Tomasz Kluz^4^, Rodney Scott^5^, Jan Lubiński^1^

#### ^1^Department of Genetics and Pathology, International Hereditary Cancer Center, Pomeranian Medical University, Szczecin, Poland; ^2^Read-Gene, Grzepnica, Dobra, Poland; ^3^Department of Urology and Urological Oncology, Pomeranian Medical University, Szczecin, Poland; ^4^Department of Gynecology, Gynecology Oncology and Obstetrics, Institute of Medical Sciences, Medical College of Rzeszow University, Rzeszow, Poland; ^5^Medical Genetics, Hunter Medical Research Institute; Priority Research Centre for Cancer Research, Innovation and Translation, School of Biomedical Sciences and Pharmacy, Faculty of Health and Medicine, University of Newcastle, Newcastle, Australia

*Hereditary Cancer in Clinical Practice* 2024, **22(1):**A11

https://www.mdpi.com/2072-6643/16/4/527

## A12 The place of genetic testing in the treatment of endometrial cancer—consequence of the TCGA Molecular Classification and the new drugs

### Radosław Mądry

#### Department of Gynecological Oncology, Poznan University of Medical Sciences, Poznan, Poland

*Hereditary Cancer in Clinical Practice* 2024, **22(1):**A12

The TCGA Molecular Classification of Endometrial Cancer in 2013 became the driving force behind changes in the diagnosis and then in the treatment of ovarian cancer. However, it was the introduction of checkpoint inhibitors and PARP inhibitors that changed the picture of endometrial cancer treatment. The RUBY, NGR-GY018, AtEND and DUO-E studies have changed the standard of treatment. It is a new challenge for physicians, geneticists and molecular biologists. The need for genetic diagnostics in this area requires the introduction of another billing product that will comprehensively qualify patients for treatment.

## A13 Serum essential elements and survival after cancer diagnosis

### Jan Lubiński^1,2^, Marcin Lener^ 1^, Wojciech Marciniak^2^, Sandra Pietrzak^1^, Róża Derkacz^2^, Cezary Cybulski^1,2^, Jacek Gronwald^1,2^, Tadeusz Dębniak^1^, Anna Jakubowska^1^, Tomasz Huzarski^1,2,3^, Milena Matuszczak^1^, Katherine Pullella^4,5^, Ping Sun^5^, Steven A Narod^5,6^

#### ^1^Department of Genetics and Pathology, International Hereditary Cancer Center, Pomeranian Medical, University in Szczecin, Szczecin, Poland; ^2^Read-Gene, Grzepnica, Dobra Poland; ^3^Department of Clinical Genetics and Pathology, University of Zielona Góra, Zielona Góra, Poland; ^4^Department of Nutritional Sciences, University of Toronto, Toronto, Canada; ^5^Women's College Research Institute, Toronto, Canada; ^6^Dalla Lana School of Public Health, University of Toronto, Toronto, Canada

*Hereditary Cancer in Clinical Practice* 2024, **22(1):**A13

https://www.mdpi.com/2072-6643/15/11/2611

## A14 c.268G > A substitution in *NTHL1* gene in Polish polyposis patients

### Andrzej Pławski

#### Institute of Human Genetics, Polish Academy of Sciences, Poznan, Poland

*Hereditary Cancer in Clinical Practice* 2024, **22(1):**A14

https://www.mdpi.com/1422-0067/24/19/14548

## A15 Epigenetic age of female *BRCA1* mutation carriers appears not to be correlated with their chronological age

### Patrycja K Przybylowicz^1^, Katarzyna E Sokolowska^1^, Olga Taryma-Leśniak^1^, Jan Bińkowski^1^, Melanie Staszewski^1^, Tomasz Huzarski^2^, Jan Lubiński^2^, Tomasz K. Wojdacz^1^

#### ^1^Independent Clinical Epigenetics Laboratory, Pomeranian Medical University in Szczecin, Szczecin, Poland; ^2^Department of Genetics and Pathology, Pomeranian Medical University in Szczecin, Szczecin, Poland

##### **Correspondence:** Tomasz K. Wojadcz, (tomasz.wojdacz@pum.edu.pl)

*Hereditary Cancer in Clinical Practice* 2024, **22(1):**A15

The changes of epigenetic age of blood cells have previously been associated with increased cancer mortality. For instance, a study on 2 029 women from WHI cohort has shown that a one-year increase of blood epigenetic age is associated with a 5% risk of lung cancer mortality (*p* = 0.031) [1]. Also, a study on 1 863 older participants from ESTHER cohort reported that increased epigenetic age of blood cells is associated with an elevated risk of any cancer mortality (HR = 1.22 [95% CI 1.03–1.45]) [2].

We hypothesized that epigenetic age acceleration observed in blood cells may be associated with *BRCA1* epimutations and germline mutations, both of which have been shown to increase risk of breast cancer.

Our study included 93 blood samples from healthy participants: 43 women with *BRCA1* germline mutation and no epimutation, 29 women with *BRCA1* epimutation and confirmed negative for germline mutations in *BRCA1* gene and 21 controls with neither *BRCA1* mutation nor epimutation present.

DNA methylation profiling of DNA extracted from whole blood samples was performed using Infinium MethylationEPIC BeadChip (Illumina). Data analysis was conducted using R 4.2.2. (IDAT processing and BMIQ normalization – ChAMP). Epigenetic age was estimated using an online tool https://dnamage.clockfoundation.org/ website and included calculation of five types of epigenetic clocks: Hannum [3], Horvath [4], skinBloodClock [5], PhenoAge [1] and GrimAge [6] (error – median absolute difference, years). Correlation between chronological and epigenetic age was assessed using linear regression. Epigenetic age acceleration was calculated using residuals from regressing epigenetic age on chronological age.

Every type of the epigenetic clock predicted epigenetic age that was coherent with chronological age for women with epimutation, as well as for women that did not carry neither epimutation or germline mutation. Moreover, women in those two groups, exhibited similar epigenetic age acceleration for all types of epigenetics clocks. However, in case of germline *BRCA1* mutation carriers neither of the epigenetic clocks, except of real-age-based GrimAge, was able to correctly predict chronological age.

In conclusion, our preliminary results indicate that *BRCA1* germline mutation carriers acquire genome-wide methylation changes that affect methylation levels at the loci used for calculation of the epigenetic age. The origins of this phenomenon are unknown and need further exploration. However, it is plausible that impaired *BRCA1* gene related DNA repair mechanisms contribute to this phenomenon.

**Funding**

This study was funded by Polish Returns grant program from Polish National Agency for Academic Exchange, grant ID: PPN/PPO/2018/1/00088/U and OPUS22 grant from National Science Centre, grant ID: 2021/43/B/NZ2/02979.

**References**

1. Levine ME, Lu AT, Quach A, Chen BH, Assimes TL, Bandinelli S, Hou L, Baccarelli AA, Stewart JD, Li Y et al: An epigenetic biomarker of aging for lifespan and healthspan. Aging (Albany NY) 2018, 10(4):573-591.
2. Perna L, Zhang Y, Mons U, Holleczek B, Saum KU, Brenner H: Epigenetic age acceleration predicts cancer, cardiovascular, and all-cause mortality in a German case cohort. Clin Epigenetics 2016, 8:64.
3. Hannum G, Guinney J, Zhao L, Zhang L, Hughes G, Sadda S, Klotzle B, Bibikova M, Fan JB, Gao Y et al: Genome-wide methylation profiles reveal quantitative views of human aging rates. Mol Cell 2013, 49(2):359-367.
4. Horvath S: DNA methylation age of human tissues and cell types. Genome Biol 2013, 14(10):R115.
5. Horvath S, Oshima J, Martin GM, Lu AT, Quach A, Cohen H, Felton S, Matsuyama M, Lowe D, Kabacik S et al: Epigenetic clock for skin and blood cells applied to Hutchinson Gilford Progeria Syndrome and ex vivo studies. Aging (Albany NY) 2018, 10(7):1758-1775.
6. Lu AT, Quach A, Wilson JG, Reiner AP, Aviv A, Raj K, Hou L, Baccarelli AA, Li Y, Stewart JD et al: DNA methylation GrimAge strongly predicts lifespan and healthspan. Aging (Albany NY) 2019, 11(2):303-327.

## A16 Are there germline genetic variants that correlate with mutational events in adult brain tumours?

### Xiajie Summer Zhang, Andrea Johns, Alexandre Xavier, Kelly Kiejda, Rodney J. Scott

#### School of Biomedical Sciences and Pharmacy, Faculty of Medicine, Health and Wellbeing, University of Newcastle, Newcastle, Australia

*Hereditary Cancer in Clinical Practice* 2024, **22(1):**A16

The search for inherited predispositions to any form of brain tumour has been hampered by difficulties in accessing tumour and blood samples from patients diagnosed with this these types of brain malignancy. The absence of material to study has resulted in little if any improvement in outcomes for the past 40 or so years. More recently, the collection of brain tumour material and constitutional DNA samples has been increasing and more information about the genetic basis of this disease is now forthcoming.

In the current study we undertook exome sequencing of 128 matched tumour and 140 constitutional DNA samples to determine if there was a genetic predisposition to brain tumours. In a first pass analysis we identified several somatic events, many of which had been previously reported, and compared these results to germline variants in an attempt to reveal any correlation between the two data sets.

The results revealed there was little if any cross over between the somatic events we identified and what was observed in the germline of this patients. We were able to correlate combinations of genes that were associated with outcome, some of which were linked to a particular poor prognosis.

In conclusion, we did not reveal any germline changes in genes that have previously linked to brain tumour susceptibility, underscoring the rarity of these variants even in a selected population of patients. This suggests that epigenetic events are more likely to be associated with adult-onset brain tumour development.

## A17 DMSA in lowering blood Pb cancerogenic level

### Ewa Siwiec^1^, Jan Lubiński^2^

#### Diagnostic Medicine of the Pomeranian Medical Universityin Szczecin, Szczecin, Poland; ^2^Department of Genetics and Pathology, Pomeranian Medical University in Szczecin, Szczecin, Poland

*Hereditary Cancer in Clinical Practice* 2024, **22(1):**A17

**Introduction**

DMSA – 2,3-dimercaptosuccinic acid is a dietary supplement (and drug) used mainly in acute Pb and Hg poisoning. It acts as a heavy metal chelator. Our previous studies showed that increased Pb levels are carcinogenic. We decided to use DMSA as a potential anti-carcinogenic agent.

**Aim of the study**

Efficiency of DMSA in lowering of blood Pb concentration

**Study groups**

1. healthy women BRCA1(-) and Pb concentration > 7,5 µg/L
2. healthy women BRCA1(+) and Pb concentration >  8,0 µg/L
3. healthy men and Pb concentration >  13,5 µg/L

**Results**

The average Pb blood concentration after 4 weeks of DMSA supplementation decreased: from 17,0 to 6,2 µg/L in BRCA1(+) group, from 14,3 to 4,6 µg/L in BRCA1(-) group and from 32,83 to 9,3 µg/L in men group. These values indicate drop of average Pb concentration by 61,1%, 66,0% and 69,3% in BRCA1(-), BRCA1(+) and men group, respectively.

**Conclusions**

DMSA may be used for effective lowering of Pb blood concentration and does not cause serious adverse effects.

## A18 Ratio of blood Zn/Cu levels as a marker of survival in breast cancer patients

### Marek Szwiec^1^, Wojciech Marciniak^2^, Róża Derkacz^2^, Tomasz Huzarski^3^, Jacek Gronwald^3^, Cezary Cybulski^3^, Tadeusz Dębniak^3^, Anna Jakubowska^3, 4^, Marcin Lener^3^, Michał Falco^5^, Józef Kładny^6^, Piotr Baszuk^3^, Jerzy Duszyński^7^, Joanne Kotsopoulos^8,9^, Steven A Narod^8,9^, Jan Lubiński^2,3^

#### ^1^Department of Surgery and Oncology, University of Zielona Góra, Zielona Góra, Poland; ^2^Read-Gene S.A., Grzepnica, Poland; ^3^Department of Genetics and Pathology, International Hereditary Cancer Center, Pomeranian Medical University, Szczecin, Poland; ^4^Independent Laboratory of Molecular Biology and Genetic Diagnostics, Pomeranian Medical University in Szczecin, Szczecin, Poland; ^5^Regional Oncology Centre, Szczecin, Poland; ^6^Department of General and Oncological Surgery, Pomeranian Medical University, Szczecin, Poland; ^7^Nencki Institute of Experimental Biology, Polish Academy of Sciences, Warsaw, Poland; ^8^Women's College Research Institute, Women's College Hospital, University of Toronto, Toronto, Canada; ^9^Dalla Lana School of Public Health, University of Toronto, Toronto, Canada

*Hereditary Cancer in Clinical Practice* 2024, **22(1):**A18

https://www.mdpi.com/2072-6643/16/7/1000.

## A19 Genomes of *BRCA1* mutation and epimutation carriers appear to acquire specific epigenetic signature

### Katarzyna Sokolowska^1^, Olga Taryma-Leśniak^1^, Jan Bińkowski^1^, Jacek Antoniewski^1^, Patrycja K Przybyłowicz^1^, Tomasz Huzarski^2^, Jan Lubiński^2^, Tomasz K. Wojdacz^1^

#### ^1^Independent Clinical Epigenetics Laboratory, Pomeranian Medical University in Szczecin, Szczecin, Poland; ^2^Department of Genetics and Pathology, Pomeranian Medical University in Szczecin, Szczecin, Poland

##### **Correspondence:** Tomasz K. Wojadcz, (tomasz.wojdacz@pum.edu.pl)

*Hereditary Cancer in Clinical Practice* 2024, **22(1):**A19

The contribution of genome wide methylation changes observed in peripheral blood of *BRCA1* epimutation and germline mutation carriers, as well as women before diagnosis of breast cancer is poorly understood. We used InfiniumMethylationEPIC BeadChip microarray technology (Illumina) and analyzed genome wide methylation patterns (over 850 000 CpG sites) in blood cells of 43 women with one of three *BRCA1* founder mutations (mean age=52.91), which were cancer free at the time of sampling and did not develop cancer within 12.9 years of follow up, 29 women with detectable in blood *BRCA1* epimutation (mean age=62.69) with 7.22 years follow up, and 19 women with neither mutation nor epimutation, which developed cancer on average 4.62 years from sampling. The controls in our experiment were 21 healthy women with neither mutation nor epimutation and cancer free follow up of 8.17 years. All samples were acquired from The International Hereditary Cancer Center (IHCC) Biobank.

In the analysis we first identified CpG sites with statistically significantly different methylation levels between women in each of the analyzed groups and controls (FDR corrected p≤0.05 methylation difference of more than 5%). We then, assessed the differences in the identified methylation signatures between groups of studied women. Each of the groups displayed different number of methylation changes, with *BRCA1* epimutation carriers harboring the highest number of 5163 differently methylated CpG sites, germline mutation carriers 2473 CpG sites, and cancer free women, who developed cancer after follow up, 12 CpG sites. This indicates that only epimutation and germline mutation carriers display increased number of genome wide methylation changes, but only a minor number of identified DMPs overlap between analyzed groups of women. Nevertheless, the biological processes identified with Gene Set Enrichment Analyses (GSEA) for epimutation and germline mutation carriers, based on genes annotated to identified methylation changes carriers were remarkably coherent. These terms included various processes occurring within mammary tissue, such as mammary gland bud morphogenesis, mammary gland specification, mammary gland bud formation, and fibroblast growth factor receptor signaling pathway, involved in mammary gland specification. Overall, our results suggest that both *BRCA1* epimutation, as well as germline mutation carriers acquire methylation changes that are not present in cancer free women and heathy women five years before cancer diagnosis, and those changes affect processes, which disruption has been shown to occur during breast cancer pathology.

**Funding**

PRELUDIUM BIS 2 grant number 2020/39/O/NZ2/02943 from the National Science Centre, Poland, Polish Returns grant program from Polish National Agency for Academic Exchange, grant ID: PPN/PPO/2018/1/00088/U and OPUS22 grant from National Science Centre, grant ID: 2021/43/B/NZ2/02979.

## A20 Genomic instability, microenvironment and telomere homeostasis in solid malignancies

### Pavel Vodička^1,2,3^, Michal Kroupa^1,3^, Kristyna Tomasova^1,3^, Anna Siskova^1,2^, Anusha Uttarilli^1^, Saba Selvi^1^, Petr Hanak^1^, Katerina Balounova^1^, Veronika Vymetalkova^1,2,3^, Sona Vodenkova^1,3^, Rajiv Kumar^1^, Kari Hemminki^3^ and Ludmila Vodickova^1,2,3^

#### ^1^Institute of Experimental Medicine, Czech Academy of Sciences, Prague, Czech Republic; ^2^Institute of Biology and Medical Genetics, First Faculty of Medicine, Charles University, Prague, Czech Republic; ^3^Biomedical Center, Faculty of Medicine in Pilsen, Charles University, Pilsen, Czech Republic

*Hereditary Cancer in Clinical Practice* 2024, **22(1):**A20

Solid tumors belong to the leading malignancies and causes of deaths worldwide. Both impaired DNA repair mechanisms and disrupted telomere length homeostasis represent key culprits in cancer initiation, progression and prognosis. Altered DNA repair results through accumulation of mutations into the genomic instability. DNA repair determines the response to chemotherapeutics in cancer treatment. Telomere attrition resulting in replicative senescence, simultaneously by-passing cell cycle checkpoints, is a hallmark of cellular malignant transformation. Telomerase is ubiquitous in advanced solid cancers and its expression is fundamental to cell immortalisation. Large-scale sequencing of human cancer samples has revealed genetic heterogeneity within individual tumors, since they are composed of diverse subpopulations/subclones variable in space and time. Aneuploidy is present in ∼80% of human solid neoplasms, the majority of which often exhibit chromosomal instability (CIN), both structural and numerical. CIN generates either abnormal aneuploid karyotypes, or continually expands phenotypic heterogeneity as tumor cell populations undergo consecutive cell divisions. Here we searched for the CIN markers in the adenoma-adenocarcinoma transition and in colorectal cancer progression, in breast and ovary cancers. Understanding the mechanisms and dynamics of tumor genomic diversification, where DNA damage response and telomere homeostasis are important players, is critical to understand carcinogenesis and overcome the drug resistance. A part of the above search is the comparison of telomere homeostasis genetics (based on GWAS study) with TL in 7,000 patients with sporadic CRC.

The mitochondrial dysfunction, another cancer hallmark, is linked with DNA repair capacity and compensate for damage by increasing the mitochondrial DNA copy number (mtDNA-CN). Current studies on the mtDNA-CN reported ambiguous and inconsistent results for various cancer types. Telomere shortening has a dual role in tumorigenesis. It promotes cancer initiation by inducing CIN, while TL maintenance characterized by telomerase expression is required for cancer cell proliferation and tumour growth. The reports on TL as a biomarker for cancer risk, patient therapy response and/or survival are contradictory as well. Our investigations were also focused on mtDNA_CN in CRC tissues and adjacent mucosa.

Acknowledgement AZV NU21-03-00145, NU21-07-00247, GAČR: 21-27902S, 21-04607X, The project National Institute for Cancer Research (Programme EXCELES, No. LX22NPO5102).

## A21 Detection of constitutional epimutation of *BRCA1* is only possible with highly sensitive method

### Filip Machaj^1^, Katarzyna Ewa Sokolowska^1^, Konrad Borowski^1^, Szymon Retfiński^1^, Dominik Strapagiel^2^, Marta Sobalska-Kwapis^2^, Tomasz Huzarski^3^, Jan Lubiński^3^, Tomasz Kazimierz Wojdacz^1*^

#### ^1^Independent Clinical Epigenetics Laboratory, Pomeranian Medical University in Szczecin, Szczecin, Poland; ^2^Biobank Laboratory, Department of Oncobiology and Epigenetics, Faculty of Biology and Environmental Protection, University of Lodz, Lodz, Poland; ^3^Department of Genetics and Pathology, Pomeranian Medical University in Szczecin, 252 Szczecin, Poland

*Hereditary Cancer in Clinical Practice* 2024, **22(1):**A21

https://www.nature.com/articles/s41598-023-43276-7.

